# The impact of low energy availability risk on pre-competition physiological function in Chinese female combat athletes

**DOI:** 10.1080/15502783.2025.2490170

**Published:** 2025-04-20

**Authors:** Yiheng Liang, Yuxuan Li, Yan Chen, Kun Meng, Fanyang Zhou, Yiran Pei, Yong Liu, Junqiang Qiu

**Affiliations:** a Beijing Sport University, Department of Exercise Biochemistry, Exercise Science School, China; b Beijing Sports Nutrition Engineering Research Center, Beijing, China; c Beijing Research Institute of Sports Science, Beijing, China

**Keywords:** Low energy availability, female athletes, combat athletes

## Abstract

**Background:**

Low energy availability (LEA) can negatively impact athletes’ physiological function and performance. This study aims to examine the prevalence of LEA in Chinese female combat athletes and monitor changes in physiological function and performance during the pre-competition period.

**Method:**

We assessed the incidence of low energy availability (LEA) and eating disorder (ED) risks in 84 female combat athletes (judo, freestyle wrestling, and sanda) from Beijing using the Low Energy Availability in Females Questionnaire (LEAF-Q) and the Eating Disorder Examination Questionnaire (EDE-Q). From this group, 11 judo athletes who were preparing for competition were selected and divided into a low energy availability (LEA) group and a non-LEA group based on their energy availability levels. Dietary intake, training energy expenditure, body composition, resting metabolic rate, blood markers, and special judo fitness tests were monitored at 4 weeks, 2 weeks, and 0 weeks before the competition.

**Results:**

Among the 84 athletes, 45.2% of athletes (*n* = 38) were at increased risk of LEA, and 21.4% of athletes (*n* = 18) were classified as high in eating disorder risk. There were no significant differences in LEA and ED risk between elite and recreational athletes. Among the 11 athletes preparing for competition, 6 athletes (45.5%) were in a state of LEA at the initial stage (4 weeks before the competition), and by the competition week, all 11 athletes exhibited LEA. Additionally, athletes in the LEA group experienced significant reductions in VO_2_ and resting metabolic rate at 0 week of the competition compared to 4 weeks prior (*p* < 0.05). Thyroid function indicators and IGF-1 levels of LEA group also significantly decreased (*p* < 0.05). After completing the four-week pre-competition weight loss, heart rate recovery during the special judo fitness test improved significantly in both the LEA and non-LEA groups (*p* < 0.05).

**Conclusion:**

The current study identified a risk of LEA among Chinese female combat sport athletes, with no significant difference in the prevalence of LEA between elite and recreational athletes. It is essential for Chinese coaches and sports medicine staff to implement LEA-related nutritional education across all performance levels. Moreover, preventive measures during training are recommended to mitigate the impact of LEA on physiological function during the pre-competition weight loss phase.

## 1. Introduction

With the continuous improvement of scientific training systems, researchers and coaches have recognized that energy availability (EA) is one of the key factors for athletes to maintain functional status and sports performance. Energy availability refers to the energy available to the body’s systems for maintaining other physiological functions after accounting for the energy expended during training [1]. Data indicate that when EA is less than 30 kcal/kg FFM/day, it can lead to short-term or long-term low energy availability (LEA) [2,3].

Pre-competition weight loss is a critical task for judo athletes during their preparation phase [4,5]. Most combat sport athletes typically choose to engage in rapid weight loss before weigh-ins, followed by rapid weight gain to restore their body weight to within 5% of their competition weight category as quickly as possible, in order to achieve optimal performance [6]. Studies have shown a positive correlation between the amount of weight lost before competition and the athletes’ performance in combat sports [7]. However, whether weight is reduced slowly or rapidly before competition, it can have certain negative effects [8,9]. Frequent weight cycling increases the risk of eating disorders (ED) and may be accompanied by LEA, thereby affecting athletes’ physiological health and performance [10,11]. However, there is still limited research on the impact of LEA caused by rapid weight loss on athletes. Additionally, further exploration is needed to determine whether the energy availability status during training exacerbates the negative effects of LEA during the RWL period.

Since 2014, the International Olympic Committee’s consensus statement on Relative Energy Deficiency in Sport (RED-S) has been updated three times, continually refining research on the potential impact of LEA on physiological function and athletic performance [10–12]. Athletes in a state of short-term or long-term LEA experience disruptions in physiological functions, including reproductive health, bone health, gastrointestinal function, energy metabolism, glucose and lipid metabolism, cardiovascular function, growth and development, immune function, and psychological health. Compared to athletes with normal energy availability EA, LEA suppresses leptin levels and is accompanied by lower plasma glucose and insulin levels [13,14]. When EA in female athletes drops below 30 kcal/kg FFM/day, it may lead to ovulatory disorders and a reduction in estradiol (E2) secretion, ultimately causing functional hypothalamic amenorrhea [2]. LEA also results in a decrease in triiodothyronine (T3) and free triiodothyronine (FT3) levels [15–17]. Due to the body’s long-term adaptive mechanisms to energy deficiency, resting metabolic rate (RMR) significantly decreases in a state of LEA [18,19]. Currently, research on LEA in female combat sport athletes is relatively limited. Therefore, this study aims to objectively evaluate the LEA status of female combat sport athletes using various indicators, providing a reference for optimizing the monitoring of physiological function during the pre-competition period.

Despite extensive research on LEA, limited studies have examined its effects during pre-competition weight loss phases in female combat athletes. This study aims to address this research gap by investigating the prevalence of LEA among Chinese female combat athletes and exploring its effects on physiological function and performance during the pre-competition period. We hope that this study will provide reference data for monitoring the physiological functions of judo athletes during the preparation phase and offer insights for coaches, researchers, and athletes in preventing the risk of LEA.

## 2. Methods

### 2.1. Participants

A total of 84 female professional athletes from combat sports (judo, freestyle wrestling, and sanda) in Beijing participated in this study. Inclusion criteria required participants to be healthy female athletes, aged over 15 years, with no history of chronic or metabolic diseases. All participants provided informed consent prior to participation and signed consent forms before testing. Athletes who had participated in national or international level competitions were classified as elite athletes (*n* = 42), while the others were categorized as recreational athletes (*n* = 42).

Among these 84 athletes, due to conflicts between the training plan coordinated by the coaching team and competition tasks, only 11 elite-level judo athletes who had participated in the Chengdu Universiade were ultimately included in the full monitoring of the pre-competition weight loss experiment. Among the 11 athletes, two competed in the −48 kg category, four in the −52 kg category, three in the −57 kg category, and one each in the −70 kg and −80 kg categories. All athletes participated in 4 to 5 competitions annually, including at least 2 national-level events. Data were collected on dietary intake, training energy expenditure, body composition, resting metabolic rate, blood biomarkers, and physical performance at different stages before competition. The current study was reviewed and approved by the Ethics Committee of the Beijing Institute of Sports Science (Approval No: TKSLL202307).

### 2.2. Study design

We conducted a questionnaire survey to assess the risk of LEA and eating disorders among female Chinese judo athletes during their regular training period. Eleven athletes preparing for competition voluntarily participated in the subsequent experiment, where their body composition, resting metabolic rate, dietary intake, training energy expenditure, blood biomarkers, and physical performance were monitored at 4 weeks, 2 weeks, and 0 weeks before the competition. These 11 athletes were divided into LEA and non-LEA groups based on their EA levels assessed at the 4-week pre-competition point, representing their long-term EA status. All athletes began rapid weight loss 4 weeks before the competition and reached their competition weight category by 0 weeks pre-competition. None achieved their target weight through acute weight loss, and no athletes aimed for weight gain before the competition. There were no differences in training content or testing protocols between the two groups at any of the pre-competition stages. The aim was to track changes in LEA-related biomarkers at different stages before the competition, while evaluating the impact of LEA status on athletes’ physiological function and performance during the pre-competition period.

### 2.3. Questionnaires

The Low Energy Availability in Females Questionnaire (LEAF-Q) was used to screen athletes for LEA risk, consistent with the questionnaire used in our previous studies [1]. A total score of ≥ 8 on the LEAF-Q indicates an increased risk of LEA, with thresholds of ≥ 2 for injury and gastrointestinal function, and ≥ 4 for menstrual disturbance. The Eating Disorder Examination Questionnaire (EDE-Q) was utilized to assess the risk of eating disorders (ED) among athletes. The EDE-Q covers four subscales: dietary restraint, eating concern, shape concern, and weight concern [20,21]. A total score of ≥ 2.3 on the EDE-Q indicates a risk of ED [22,23].

### 2.4. Body composition

Athletes were required to wear minimal clothing, and their fasting body weight was measured using a body composition analyzer (InBody720, Korea). Body composition was assessed using dual-energy X-ray absorptiometry ((Lunar iDXA, GE Healthcare, Madison, WI, USA). All measurements were conducted in the morning with the athletes in a fasting state. Before the test, the athletes lay in the standard anatomical position on the testing table, and their knee and ankle joints were secured by the staff. The entire testing procedure took approximately 10 minutes.

### 2.5. Resting metabolic rate

Resting metabolic rate (RMR) was measured at 4 weeks and 0 weeks before the competition using a gas metabolism analyzer (Highermed Smax58ce, Hanyan, China) and Polar H10 sensor chest strap device (Polar Electro Oy, Kempele, Finland). In the morning, while awake, fasting, and in a quiet state, the athletes wore the gas metabolism analyzer breathing mask and the heart rate monitor. Once the measurements were stable, resting gas metabolism data were recorded. The actual resting metabolic rate (RMR) was calculated using the Weir equation [24], while the predicted RMR was calculated using the Cunningham equation [25]. The resting metabolic ratio (RMRratio) was defined as the ratio of the measured RMR to the predicted RMR [26].

### 2.6. Dietary survey and training energy expenditure

Energy intake (EI) was continuously recorded for 2 training days and 1 rest day using the food weighing method. Energy expenditure (EE) during all training sessions on the 2 training days and 1 rest day was monitored using heart rate measurements. Exercise energy expenditure (EEE) was calculated based on the actual heart rate during training. The formula for EEE (kcal/kg/min) is: EEE (kcal/kg/min)=((5.95*HRaS) + (0.23*age) + (84 × 1)–134)/4186.8 [27,28]. where age represents the athlete’s age, and HRaS is the average heart rate above the sleeping heart rate. The sleeping heart rate is calculated as 0.83 times the heart rate measured during the RMR test.

Energy availability (EA) were calculated based on dietary intake, training energy expenditure, and body composition data. An EA value of ≥30 kcal/kg FFM (fat-free mass)/day indicates that the athlete is not in a state of LEA, while an EA value of <30 kcal/kg FFM/day indicates that the athlete is in a state of LEA.

### 2.7. Blood tests

Venous blood samples were collected from the athletes’ elbows in the morning. The blood biomarkers measured included Thyroid Stimulating Hormone (TSH), triiodothyronine (T3), free triiodothyronine (FT3), thyroxine (T4), free thyroxine (FT4), insulin-like growth factor 1 (IGF-1), estradiol (E2). IGF-1 were measured using ELISA kits (Elabscience, China). TSH, T3, FT3, T4, FT4 and E2 were measured using a fully automated biochemical analyzer (Mindray, China).

### 2.8. Special judo fitness test

Special Judo Fitness Test (SJFT) was conducted 4 weeks and 0 weeks before the competition. Prior to the test, athletes were grouped according to their weight categories, with the grouping and testing order remaining consistent across both sessions. SJFT consists of three phases: Phase 1 (15 seconds), Phase 2 (30 seconds), and Phase 3 (30 seconds), with a 10-second rest interval between each phase. At the start of each phase, the athletes were required to run toward two partners positioned 6 meters apart and perform as many ippon-seoi-nage throws as possible within the allotted time. The number of successful throws (Nb) was recorded, along with the heart rate immediately after the test (HR1) and one minute after exercise (HR2). The results were used to calculate the SJFT Index (SJFT Index = (HR1 + hR2)/Nb), which was used to assess the athletes’ performance [29].

## 3. Statistical analyses

All data were analyzed using SPSS 21.0. Parametric data were reported as mean ± standard deviation, whereas nonparametric data were presented as median with interquartile range (25th and 75th percentiles). The Shapiro-Wilk test was conducted to assess the normality of the data. To determine the differences between EA status (high-risk and low-risk) and athletic levels (elite athletes and recreational athletes), an independent samples t-test was used for parametric data analysis, while the Mann-Whitney U test was applied for non-parametric data. Chi-square tests were conducted to assess differences in the incidence of LEA, ED, injuries, gastrointestinal dysfunction, and menstrual disturbances between the groups. Two-way repeated measures ANOVA was performed to examine the interaction between group (LEA and non-LEA) and assessment time (4 weeks, 2 weeks, and 0 weeks before the competition). Where significant differences were identified, post-hoc Bonferroni multiple comparison tests were applied. The significance level for all statistical tests was set at *p* < 0.05.

## 4. Result

### 4.1. Risk of LEA in Chinese female combat athletes

Elite athletes were significantly older, had a higher BMI, and reported more years of training, higher training frequency, and greater weekly exercise duration. Although elite athletes had higher LEAF-Q scores, the difference was not statistically significant. There were no significant differences in height, weight, or EDE-Q scores between the two groups (Table 1).

**Table 1.** Participants characteristics.

	Elite athletes (n = 42)	Recreational athletes (n = 42)	*p*-value
Age (y)	20 ± 3	17 ± 2	<0.001*
Height (cm)	166.0 ± 6.6	166.3 ± 4.8	0.851
Weight (kg)	65.0 ± 15.5	60.3 ± 8.1	0.088
BMI (kg/m^2^)	23.3 ± 3.6	21.8 ± 2.3	0.021*
Years of training (y)	7.0 (5.4–10.0)	5.0 (4.0–5.0)	<0.001*
Frequency per week (d)	6.0 (4.9–6.0)	5.0 (3.0–5.9)	0.002*
Exercise per week (h)	35.5 (29.5–36.0)	30.0 (21.0–36.0)	0.023*
LEAF-Q score	8.0 (5.8–10.0)	7.0 (5.0–8.0)	0.062
EDE-Q score	1.3 (0.5–2.1)	1.2 (0.4–2.3)	0.806

*indicates *p* < 0.05.

The comparison between elite athletes and recreational athletes revealed a significantly higher injury incidence among elite athletes. Although the prevalence of LEA risk was higher in elite athletes and the occurrence of secondary amenorrhea was more frequent, neither difference reached statistical significance. There were no significant differences between the two groups in terms of ED risk, gastrointestinal dysfunction, menstrual disturbances, primary amenorrhea, or secondary amenorrhea (Table 2).

**Table 2.** Prevalence of physiological symptoms in elite vs. recreational athletes.

	Elite athletes (n = 42)	Recreational athletes (n = 42)	χ^2^	*p*-value
	n (%)	n (%)		
LEA risk	22 (52.4%)	16 (38.1%)	1.730	0.273
ED risk	8 (19.0%)	10 (23.8%)	2.472	0.291
Injury	23 (54.8%)	8 (19.0%)	11.503	0.001*
Gastrointestinal dysfunction	22 (52.4%)	22 (52.4%)	<0.001	1.000
Menstrual disturbance	25 (59.5%)	23 (54.8%)	0.194	0.826
Primary amenorrhea	3 (7.1%)	3 (7.1%)	<0.001	1.000
Secondary amenorrhea	18 (42.9%)	12 (28.6%)	1.867	0.255

*indicates *p* < 0.05.

The comparison between athletes at risk of LEA (LEAF-Q total score ≥ 8, *n* = 38, 45%) and those not at risk (LEAF-Q total score < 8, *n* = 46, 55%) showed no significant differences in anthropometric and training profile. However, athletes at risk of LEA had significantly higher LEAF-Q scores and showed a trend toward longer weekly training hours. No significant difference was found in EDE-Q scores between the two groups (Table 3).

**Table 3.** Athlete characteristics by LEA risk classification.

	Not at risk of LEA (n = 46)	At risk of LEA (n = 38)	*p*-value
Age (y)	18 ± 3.4	19 ± 3.0	0.143
Height (cm)	167.4 ± 5.8	164.7 ± 5.5	0.114
Weight (kg)	63.0 (55.0–70.8)	59.3 (54.7–65)	0.268
BMI (kg/m^2^)	22.8 (20.0–24.0)	22.0 (20.9–23.1)	0.593
Years of training (y)	6.3 (4.1–7.0)	6.4 (4.0–8.3)	0.884
Frequency per week (d)	6.0 (6.0–7.0)	6.0 (6.0–7.0)	0.456
Exercise per week (h)	30.0 (21.0–36.0)	35.0 (30.0–36.0)	0.061*
LEAF-Q score	5.0 (4.0–6.8)	9.5 (8.0–12.0)	<0.001*
EDE-Q score	1.1 (0.4–2.1)	1.4 (0.7–2.6)	0.118

*indicates *p* < 0.05.

The comparison between athletes at risk of LEA and those not at risk revealed significantly higher rates of injury, gastrointestinal dysfunction, and menstrual disturbances in the LEA risk group. Additionally, the prevalence of primary amenorrhea and secondary amenorrhea was significantly higher in the LEA risk group. Although the risk of ED was higher in the LEA risk group, this difference was not statistically significant (Table 4).

**Table 4.** Prevalence of physiological symptoms by LEA risk status.

	Not at risk of LEA (n=46)	At risk of LEA (n=38)	χ^2^	*P*-value
	n (%)	n (%)		
ED risk	7 (15.2%)	11 (28.9%)	3.723	0.155
Injury	11 (23.9%)	20 (52.6%)	7.371	0.012*
Gastrointestinal dysfunction	17 (37.0%)	27 (71.1%)	9.699	0.002*
Menstrual disturbance	15 (32.6%)	33 (86.8%)	24.993	<0.001*
Primary amenorrhea	0 (0%)	6 (15.8%)	7.822	0.007*
Secondary amenorrhea	7 (15.2%)	23 (60.5%)	18.607	<0.001*

*indicates *p* < 0.05.

### 4.2. Energy availability across pre-competition stages

There were no significant differences in macronutrient distribution between the non-LEA and LEA groups at different pre-competition stages. Overall, the carbohydrate, protein, and fat intake proportions were approximately 40%, 20%, and 40% in two group. In the non-LEA group, carbohydrate intake at 4 weeks before the competition was significantly higher than at 0 weeks. Fat intake at 4 weeks before the competition was also significantly higher than at 2 weeks and 0 weeks before the competition. Additionally, in the non-LEA group, fat intake at 4 weeks before the competition was significantly higher than in the LEA group. The pre-competition protein intake was approximately 1.81 g/kg in the non-LEA group and 1.47 g/kg in the LEA group, with no significant difference between the two groups.

At 4 weeks before the competition, 6 athletes (54.5%) were in a state of LEA; at 2 weeks before the competition, 9 athletes (81.8%) were in a state of LEA; and by 0 weeks before the competition, all 11 athletes (100%) were experiencing LEA.

At 4 weeks before the competition, the non-LEA group had significantly higher EI and EA levels compared to the LEA group (*p* = 0.041; *p* = 0.006, respectively). In the non-LEA group, EI at 0 weeks before the competition was significantly lower than at 4 weeks and 2 weeks (*p* = 0.004; *p* = 0.028, respectively), with EI at 4 weeks also being higher than at 2 weeks(*p* = 0.009). In the LEA group, only EI at 4 weeks before the competition was significantly higher than at 0 weeks(*p* = 0.048). Both groups exhibited a significant decrease in EEE levels at 0 weeks compared to 4 weeks before the competition (*p*<0.05). Additionally, in the non-LEA group, EA levels at 4 weeks were significantly higher than at 2 weeks and 0 weeks before the competition (*p*<0.01; *p* = 0.012, respectively), while in the LEA group, EA levels showed no significant differences across these time points (Table 5).

**Table 5.** Macronutrient intake composition in non-LEA and LEA groups at different pre-competition stages.

	Time	Non-LEA	LEA
Carbohydrate (%)	4WPC	38.36 ± 6.06	44.38 ± 5.89
	2WPC	44.67 ± 9.73	43.91 ± 5.01
	0WPC	41.83 ± 11.41	46.12 ± 1.41
Protein (%)	4WPC	19.55 ± 3.34	19.39 ± 1.76
	2WPC	20.04 ± 3.97	21.77 ± 4.29
	0WPC	22.37 ± 3.67	21.03 ± 4.62
Fat (%)	4WPC	42.1 ± 3.33	36.23 ± 6.47
	2WPC	35.28 ± 7.55	34.32 ± 0.76
	0WPC	35.79 ± 12.91	32.85 ± 4.96
Carbohydrate intake (g/kg)	4WPC	4.28 ± 1.24	3.53 ± 0.79
	2WPC	4.1 ± 1.25	3.37 ± 0.92
	0WPC	2.63 ± 0.79^a^	2.59 ± 0.62
Protein intake (g/kg)	4WPC	2.17 ± 0.55	1.54 ± 0.28
	2WPC	1.81 ± 0.41	1.64 ± 0.36
	0WPC	1.44 ± 0.45	1.17 ± 0.30
Fat intake (g/kg)	4WPC	2.06 ± 0.28	1.28 ± 0.33*
	2WPC	1.44 ± 0.40^a^	1.16 ± 0.23
	0WPC	1.03 ± 0.53^a^	0.82 ± 0.23

WPC represents the number of weeks pre-competition. *indicates *p* < 0.05 compared to the non-LEA; ^a^indicates *p* < 0.05 compared to 4WPC.

### 4.3. Body weight and composition across pre-competition stages

There was a significant time effect on body weight and body fat percentage in both the non-LEA and LEA groups before the competition (*p* = 0.04; *p* < 0.001, respectively), but no significant time_*_group interaction was observed (*p* = 0.87; *p* = 0.52, respectively). However, no significant differences in total muscle mass were observed among the different pre-competition stages within either the non-LEA or LEA groups (Table 6).

**Table 6.** Pre-competition changes in EI, EE, and EA.

Time		Non-LEA (n = 5)	LEA (n = 6)	Total (n = 11)	LEA n (%)
4WPC	EI (kcal/day)	2596.4 ± 627.7	1919.8 ± 242.1*	2190.4 ± 534.8	
	EEE (kcal/day)	1290.7 ± 335.8	1289.1 ± 277.6	1289.7 ± 283.6	
	EA (kcal/kg FFM/day)	39.0 ± 9.7	18.2 ± 8.1*	26.5 ± 13.5	6 (54.5%)
2WPC	EI (kcal/day)	2042.8 ± 379.9^a^	1706.28 ± 424.6	1840.9 ± 422.5	
	EEE (kcal/day)	1203.8 ± 117.9	1289.3 ± 189.8	1255.1 ± 163.1	
	EA (kcal/kg/FFM/day)	19.9 ± 9.2^a^	15.4 ± 6.4	17.2 ± 7.5	9 (81.8%)
0WPC	EI (kcal/day)	1406.2 ± 319.3^ab^	1347.7 ± 248.8^a^	1371.1 ± 263.2	
	EEE (kcal/day)	913.5 ± 106.7^ab^	861.8 ± 172.8^ab^	882.5 ± 145.2	
	EA (kcal/kg/FFM/day)	13.6 ± 9.8a	11.7 ± 3.2	12.5 ± 6.2	11 (100%)

WPC represents the number of weeks pre-competition. *indicates *p* < 0.05 compared to the non-LEA; ^a^indicates *p* < 0.05 compared to 4WPC; ^b^indicates *p* < 0.05 compared to 2WPC.

### 4.4. Changes in resting metabolic rate pre-competition

Table 2 showed that in the LEA group, VO_2_, measured RMR, and RMRratio at 4 weeks before the competition were all significantly lower than at 0 weeks before the competition (*p* -values of 0.003, 0.004, and 0.004, respectively). In contrast, the non-LEA group showed no significant differences in resting metabolic rate-related data across the different pre-competition stages. Furthermore, no time*group interaction was observed for VO_2_, measured RMR, predicted RMR, or RMRratio (Table 7).

**Table 7.** Changes in body weight and body composition across different pre-competition stages.

	Time	Non-LEA	LEA
weight (kg)	4WPC	57.5 ± 5.5	62.3 ± 10.4
	2WPC	56.3 ± 4.3^a^	61.2 ± 10.1^a^
	0WPC	55.0 ± 4.6^ab^	60.0 ± 4.8^ab^
Body fat percentage (%)	4WPC	22.3 ± 1.7	23.0 ± 4.8
	2WPC	21.2 ± 2.1^a^	21.6 ± 4.7^a^
	0WPC	19.6 ± 1.7^ab^	20.2 ± 4.7^ab^
total muscle mass (kg)	4WPC	42.88 ± 4.40	45.69 ± 4.70
	2WPC	42.67 ± 4.41	45.72 ± 4.92
	0WPC	42.50 ± 4.33	45.24 ± 5.89

WPC represents the number of weeks pre-competition. ^a^indicates *p* < 0.05 compared to 4WPC; ^b^indicates *p* < 0.05 compared to 2WPC.

### 4.5. Biomarkers for energy deficiency across different pre-competition stages

In the LEA group, T3(*p* = 0.007), FT3(*p* = 0.046), T4(*p* = 0.22) and FT4(*p* = 0.006) levels at 4 weeks before the competition were higher compared to 0 weeks before the competition. Similarly, at 2 weeks before the competition, T3(*p* = 0.015) and FT3(*p* = 0.035) levels were also higher than those at 0 weeks. However, in the non-LEA group, no significant differences were observed in thyroid function markers across the different pre-competition stages. Additionally, no significant differences in thyroid function data were found between the two groups at any pre-competition stage (*p* > 0.05).

In the LEA group, IGF-1 levels at 0 weeks before the competition were significantly lower than at 4 weeks (*p* = 0.017) and 2 weeks (*p* = 0.045) before the competition. However, in the non-LEA group, there were no significant differences in IGF-1 levels across the different pre-competition stages (*p*>0.05). Additionally, at 0 weeks before the competition, IGF-1 levels in the LEA group were significantly lower than in the non-LEA group. For E2, there were no significant differences between the LEA and non-LEA groups across the different pre-competition stages, and no time*group interaction was observed (Tables 8, 9 and 10).

**Table 8.** Changes in resting metabolic rate pre-competition.

	Time	Non-LEA	LEA
VO_2_ (ml/h)	4WPC	224.3 ± 20.7	220.5 ± 31.0
	0WPC	216.1 ± 19.4	196.1 ± 30.7a
Measured RMR (kcal/day)	4WPC	1528.6 ± 147.3	1491.7 ± 210.7
	0WPC	1456.2 ± 126.6	1334.2 ± 223.2^a^
Predicted RMR (kcal/day)	4WPC	1443.2 ± 96.6	1505.2 ± 103.3
	0WPC	1484.5 ± 167.9	1495.2 ± 129.6
RMRratio	4WPC	1.07 ± 0.17	0.99 ± 0.07
	0WPC	1.00 ± 0.18	0.89 ± 0.07^a^

WPC represents the number of weeks pre-competition. ^a^indicates p  < 0.05 compared to 4WPC;

**Table 9.** Changes in biomarkers for energy deficiency across different pre-competition stages.

	Time	Non-LEA	LEA
TSH (mIU/L)	4WPC	1.83 ± 0.40	1.52 ± 0.25
	2WPC	1.65 ± 0.41	1.32 ± 0.47
	0WPC	1.91 ± 0.94	1.85 ± 0.56
T3 (nmol/L)	4WPC	1.58 ± 0.12	1.66 ± 0.27
	2WPC	1.37 ± 0.24	1.43 ± 0.43
	0WPC	1.21 ± 0.21	1.14 ± 0.51^ab^
FT3(pmol/L)	4WPC	5.08 ± 0.33	5.01 ± 0.52
	2WPC	4.61 ± 0.71	4.70 ± 1.14
	0WPC	4.25 ± 0.56	4.10 ± 0.93^ab^
T4 (nmol/L)	4WPC	80.7 ± 7.4	84.3 ± 16.6
	2WPC	80.8 ± 10.4	80.8 ± 19.1
	0WPC	78.2 ± 7.0	80.0 ± 18.1^a^
FT4(pmol/L)	4WPC	16.6 ± 0.9	16.8 ± 2.2
	2WPC	16.9 ± 2.2	16.5 ± 3.2
	0WPC	15.9 ± 1.5	15.1 ± 2.7^a^
IGF-1 (ng/ml)	4WPC	2.0 ± 0.8	2.1 ± 1.1
	2WPC	1.5 ± 0.8	1.1 ± 0.7^a^
	0WPC	1.9 ± 0.6	1.1 ± 0.5^a^
E2 (pmol/L)	4WPC	67.3 ± 30.9	53.2 ± 20.4
	2WPC	57.6 ± 25.0	48.7 ± 26.0
	0WPC	65.1 ± 32.2	54.3 ± 20.2

WPC represents the number of weeks pre-competition. ^a^indicates *p* < 0.05 compared to 4WPC; ^b^indicates *p* < 0.05 compared to 2WPC; *indicates *p* < 0.05 compared to non-LEA.

**Table 10.** Changes in performance across different pre-competition stages.

	Time	Non-LEA	LEA
successful throws (Nb)	4WPC	26.7 ± 1.2	26.0 ± 1.5
	0WPC	24.7 ± 2.5	25.5 ± 1.4
HR_1_ (bpm)	4WPC	180.7 ± 11.9	178.7 ± 9.2
	0WPC	171.0 ± 14.8a	166.2 ± 6.8a
HR_2_ (bpm)	4WPC	157.7 ± 5.5	153.3 ± 12.1
	0WPC	138.0 ± 17.1a	126.0 ± 11.7a
Index	4WPC	12.7 ± 0.5	12.8 ± 1.3
	0WPC	12.5 ± 0.8	11.5 ± 1.2a

### 4.6. Performance across different pre-competition stages

In both the non-LEA and LEA groups, HR_1_ (*p*  = 0.008) and HR_2_ (*p* < 0.001) at 4 weeks before the competition were significantly higher than at 0 weeks. Additionally, the Index in the LEA group was significantly lower at 0 weeks compared to 4 weeks before the competition. However, there were no significant differences in performance indicators between the non-LEA and LEA groups across the different pre-competition stages (*p*  > 0.05).

WPC represents the number of weeks pre-competition. a indicates *p* < 0.05 compared to 4WPC.

## 5. Discussion

The current study is the first to investigate the risk of LEA among female athletes in combat sports in China. The results revealed that 38 out of 84 athletes (45%) were at risk of LEA. This finding is similar to a previous study on 166 Chinese aesthetic sports athletes, where 41.6% were identified as at risk of LEA [30]. Similar LEA risk rates were also observed in studies involving 833 Irish female athletes and 317 elite Australian athletes who competed in the 2016 Rio Olympics, with 39.7% and 40% of female athletes at risk of LEA, respectively [31,32]. However, studies on elite Norwegian female soccer players and Polish female canoe athletes reported lower LEA risk rates of 32% and 15%, respectively [33,34]. These differences could be attributed to variations in the nationality and sport types of the athletes involved.

Eating disorders are widely recognized as one of the key contributors to LEA [35]. In this study, 18 out of 84 female combat sport athletes (21.4%) were identified as being at risk of ED. Similarly, a study by Elin et al. on 85 female runners found that 15 athletes (17.6%) were at risk of ED [23]. However, a study by Dimitra et al. on elite female basketball, volleyball, and water polo athletes in Greece reported a much lower ED risk, with only 6.2% of athletes being affected, and water polo athletes showing the highest risk [36]. These findings align with the consensus statement from The Australian Institute of Sport, which suggests that weight-class and endurance athletes are at a higher risk of ED [35].

The current study observed that athletes at risk of LEA engaged in 5 additional training hours per week compared to those not at risk. This is consistent with findings from Danielle and Slater et al., where it was observed that an additional hour of training per week increased the risk of LEA by 1.06 to 1.13 times [31,37]. Although disordered eating behaviors and ED are widely regarded as primary contributors to LEA in athletes [35], the current study found no significant difference in ED risk between athletes with low and high LEA risk. This contrasts with previous research findings [38,39], possibly because combat sport athletes tend to engage in rapid weight reduction before competitions, while maintaining stable weight during regular training periods. Additionally, while prior research has indicated a higher incidence of LEA among elite athletes [30,31], this study found no significant differences in LEA risk, ED risk, or related physiological outcomes between elite and recreational athletes, except for a higher incidence of injury in the elite group. This study did not identify any significant differences, which may be attributed to the fact that all athletes were from the Beijing team and followed a relatively uniform training program. Additionally, it may also be due to differences in weight management methods among different combat sports.

Due to the small sample size in the pre-competition monitoring phase, the results of this study’s pre-competition monitoring should be interpreted with caution and validated in larger cohorts, but should not be disregarded. The current study examined EA during different pre-competition stages, revealing that the two groups of athletes had significantly different EA levels at 4 weeks before the competition. As the competition approached, athletes adhered to weight-loss strategies, resulting in a decrease in EI. Previous case studies have found that EA decreases significantly during the weight loss period, with an average EA of approximately 20 kcal/kg FFM/day throughout the entire period, while in the week before the competition, EA can drop to −4 to 9 kcal/kg FFM/day [40,41]. In our study, the EA of all athletes also showed a gradual decline. However, since no acute weight loss was performed, their EA in the week before the competition remained at approximately 12 kcal/kg FFM/day. At 0 weeks before the competition, EE had also significantly decreased, as athletes aimed to reach their prime performance state for the event. An assessment of EA across pre-competition stages showed that 6 athletes (54.5%) were in a state of LEA at 4 weeks before the competition, reflecting their regular training phase. This aligns with previous research that monitored EA in young athletes, where 50.8% of female athletes across various sports were found to be in a state of LEA [17]. In this study, 9 athletes (81.8%) were in a state of LEA at 2 weeks before the competition, and by 0 weeks, all athletes were in a state of LEA. This pattern is closely tied to the weight-loss goals that judo athletes must achieve before competition. Similar findings have been observed in athletes from other sports requiring pre-competition weight reduction [42,43].

During the weight-loss phase, athletes experienced significant reductions in body weight and body fat percentage while largely maintaining muscle mass. This is consistent with findings by Carl et al., who studied pre-competition weight reduction in mixed martial arts athletes [41].The macronutrient distribution in all athletes’ diets remained relatively stable during the pre-competition phase. Due to the pre-competition weight reduction plan, the non-LEA group had significantly higher fat and carbohydrate intake at 4 weeks before the competition. However, athletes in the LEA group may have already been in a state of low energy availability, resulting in no significant changes in nutrient intake across the pre-competition stages. Additionally, fat intake in the non-LEA group at 4 weeks before the competition was significantly higher than in the LEA group. This may be related to the cooking methods of Chinese cuisine and the relatively high fat content in the diet [44]. With the implementation of stricter dietary control before the competition, fat intake in all athletes gradually decreased. Previous studies have indicated that even under energy deficiency, 2–3 times the RDA protein intake combined with strength training can maintain or even increase muscle mass [40,42,43]. Although the protein intake in both groups was below 2 g/kg body weight during the pre-competition period, all athletes consistently competed in a fixed weight category according to the coaching staff’s plan. This factor may explain why muscle mass did not show a significant decrease during the weight loss period. The ability to preserve muscle mass during this period may also be attributed to the athletes’ training regimen, particularly strength-specific exercises.

In the LEA group, VO_2_, measured RMR, and RMRratio all exhibited significant declines at 0 weeks before the competition, consistent with findings from previous research on weight reduction in athletes [40]. The longer the period of low dietary intake and the greater the extent of weight loss, the more pronounced the reduction in RMR may become. Some studies have indicated that an RMRratio below 0.90 may be a reliable indicator of an athlete being in a state of LEA [45]. In this study, the RMRratio in the LEA group decreased from 0.99 to 0.89, a result that aligns with findings from prior research [41,46]. Conversely, in the non-LEA group, even though EA levels dropped below 30 kcal/kg FFM/day before the competition, no significant changes in RMR were observed. This lack of change could be attributed to the relatively short duration of the LEA state, which may not have been sufficient to induce symptoms associated with Relative Energy Deficiency in Sport (RED-S) [47].

The results of LEA-related biomarkers at different pre-competition stages showed that in the LEA group, levels of T3, FT3, T4, and FT4 all significantly decreased at 0 weeks before the competition. Similar findings were reported in studies, where T3 levels significantly declined during pre-competition weight loss and failed to return to baseline even one month post-competition [48–50]. It has been suggested that a decline in thyroid function is associated with a decrease in energy expenditure, which may explain why athletes in the LEA group experienced a significant reduction in resting metabolic rate despite no apparent decrease in skeletal muscle mass [51]. TSH did not show significant changes at different pre-competition time points, but at 0 weeks before the competition, it showed an upward trend, which may be related to the decrease in T3 and T4 levels as well as the reduced training load of athletes before the competition. We also observed a significant decrease in IGF-1 levels in the LEA group at 0 weeks before the competition. Previous studies have similarly found a marked decline in IGF-1 levels in combat sport athletes during an 8-week weight loss training period [41]. However, in studies on male athletes, no significant changes in IGF-1 levels were observed during a LEA state, possibly because the LEA condition lasted only 4 days [52]. At 0 weeks before the competition, IGF-1 levels in the LEA group were significantly lower than those in the non-LEA group, which may be related to the duration of inadequate EA and the training conducted while in a LEA state [53]. In a study by Meng et al. on the risk of LEA in elite athletes participating in weight-sensitive sports, it was found that athletes at high risk of LEA had significantly lower E2 levels compared to healthy athletes [30]. Since E2 is influenced by the menstrual cycle and this study was conducted during the athletes’ pre-competition period, we cannot exclude this factor, which may explain why no significant differences in E2 levels were observed in our study.

After pre-competition training, both the non-LEA and LEA groups showed significant reductions in HR1 and HR2 at 0 weeks before the competition, and there was no difference in the number of repetitions completed in the specific test. This indicates that the athletes’ aerobic capacity and sport-specific fitness improved during the pre-competition period [29]. Similarly, male mixed martial arts athletes undergoing an 8-week weight loss training period under low EA conditions showed an upward trend in strength test performance and maximal oxygen uptake [41]. In a study where cyclists maintained constant EI while gradually increasing training load over 6 weeks, significant improvements in aerobic capacity were also observed [54]. The SJFT index decreased in the LEA group, while it remained relatively unchanged in the non-LEA group, which may be attributed to the athletes’ increased focus on specific skill training during the pre-competition phase. During the pre-competition preparation phase, judo athletes not only increase their training load but also supplement their sport-specific technical training with additional strength circuit training and specific physical conditioning. This combination likely enables athletes to maintain the same number of ippon-seoi-nage throws while achieving a lower heart rate.

## 6. Conclusion

Our study data indicate a high prevalence of LEA among Chinese female combat sport athletes. Aside from injury, there were no differences between elite and recreational athletes in terms of LEA risk, eating disorder risk, gastrointestinal dysfunction, or menstrual disturbances. We recommend enhancing LEA-related nutritional education for combat sport athletes across different performance levels by integrating regular energy availability assessments and individualized dietary interventions into training programs to prevent LEA. Our study also indicates that elite female judo athletes experience varying degrees and durations of LEA during the pre-competition preparation phase as they strive to achieve their weight loss goals. Furthermore, female judo athletes who are already in a state of LEA during regular training tend to exhibit more pronounced fluctuations in resting metabolic rate and LEA-related biomarkers following pre-competition weight loss. Athletes are advised to maintain adequate dietary intake during regular training to prevent low energy availability (LEA) and ensure optimal condition before competition.

## 7. Limitations and strengths

The study aimed to document LEA-related data during the weight reduction period of elite female judo athletes preparing for national-level competitions, with the goal of enabling athletes to safely and effectively manage pre-competition weight loss while mitigating the risks associated with LEA. Given the focus on elite athletes competing in national-level events, the sample size in this study was relatively small. Future research should involve a larger cohort of athletes to better understand the physiological changes that occur during the pre-competition phase. On the other hand, due to the significant differences in weight-cutting methods across combat sports, the risk factors for LEA in each sport should be further examined. Meanwhile, exploring individualized weight-cutting strategies in greater depth may help reduce the risk of LEA in combat athletes. Additionally, the study was conducted in alignment with the athletes’ and coaches’ actual training schedules, which limited our ability to gather post-competition recovery data. Future research should further assess post-competition recovery to gain deeper insights into the long-term impact of LEA on combat sport athletes, as well as the reversibility of these effects and the time required for recovery. Given that judo athletes experience weight cycling throughout the year, longitudinal studies are recommended to track changes in LEA across multiple training cycles and further evaluate whether the severity and duration of LEA may cause long-term damage to physiological function.
